# Knowledge, attitudes, and practices regarding nasopharyngeal carcinoma among young adults and students in southern China: a cross-sectional study

**DOI:** 10.3389/fonc.2026.1776276

**Published:** 2026-03-19

**Authors:** Yehua Dai, Meidan Cai, Dingke Liu, Ting Wang, Linzhi Kang, Ruijie Yang, Rui Huang, Liping Jiang, Liping Qi, Chunhong Shi

**Affiliations:** 1 School of Nursing, Xiangnan University, Chenzhou, Hunan, China; 2 Department of Radiation Oncology, the First People's Hospital of Chenzhou City, Chenzhou, Hunan, China; 3 Department of Radiation Oncology, Cancer Center, Sun Yat-sen University, Guangzhou, China

**Keywords:** attitude, China, cross-sectional studies, health behavior, knowledge, nasopharyngeal neoplasms, surveys and questionnaires, young adult

## Abstract

**Background:**

This study aimed to evaluate the knowledge, attitudes, and practices (KAP) related to nasopharyngeal carcinoma (NPC) among young adults and students, a key subgroup of the general population, in Southern China.

**Methods:**

A cross-sectional survey was conducted between February 6 and March 5, 2025, targeting individuals from the general population in southern China. Structural equation modeling (SEM) analysis was employed to examine the direct and indirect relationships among KAP dimensions.

**Results:**

A total of 664 valid questionnaires were obtained. Among the respondents, 421 (63.4%) were female, and 335 (50.5%) identified as students. The knowledge, attitude, and practice scores were 7.99 ± 3.80 (possible range: 0–15), 34.45 ± 4.85 (possible range: 8–40), and 43.88 ± 7.30 (possible range: 10–50), respectively. SEM analysis demonstrated that knowledge had a significant direct effect on attitude (β = 0.329, P < 0.001), while attitude had a strong direct effect on practice (β = 0.866, P < 0.001). Although the direct effect of knowledge on practice was not statistically significant (β = 0.013, P = 0.641), an indirect effect of knowledge on practice through attitude was observed (β = 0.285, P < 0.001).

**Conclusion:**

Young adults and students in Southern China exhibit low knowledge of NPC but demonstrates positive attitudes and practices. Future targeted health education interventions could enhance public knowledge, thereby further strengthening positive attitudes and promoting more effective preventive behaviors in clinical and community settings.

## Introduction

Nasopharyngeal carcinoma (NPC), a malignant tumor of the nasopharyngeal epithelium, is a prevalent head and neck cancer in endemic regions. In 2020, approximately 133,354 new NPC cases were reported globally, representing 0.7% of all cancer diagnoses, with 85.2% occurring in Asia (1). The disease exhibits a marked geographic variation, with Southern China facing a particularly high burden. For example, the incidence rate among males is 0.5 per 100,000 in northern China but reaches 25 per 100,000 in Guangdong Province (2). The elevated incidence in Southern China is attributed to genetic, viral, and environmental factors. Additionally, a notable gender disparity exists, with males having approximately three times the incidence of females, potentially due to genetic or behavioral factors (3).

The epidemiology of NPC in southern China is influenced by unique cultural and lifestyle factors, with diet and healthcare access significantly shaping disease patterns (4). While Epstein-Barr virus (EBV) infection is the predominant oncogenic factor in endemic areas—91.9% of NPC cases are EBV-positive compared to 7.7% HPV-positive (5), environmental and early-life exposures also drive regional differences in incidence. Declining NPC rates in economically advanced regions reflect shifting lifestyles and increased risk awareness (6). Early detection significantly improves NPC outcomes, with Stage I patients achieving a 100% 5-year survival rate, compared to 67.2% for Stage IV diagnoses (7). However, the tumor’s deep anatomical location and lack of distinct early symptoms often result in delayed diagnosis—averaging 23.5 weeks from symptom onset—and a 43.4% misdiagnosis rate (8, 9). Current screening in high-risk areas relies on EBV-related biomarkers, including serological antibody tests and EBV-DNA analysis, but these methods are hindered by low positive predictive values (typically <10%), despite adequate sensitivity and specificity (10). These limitations underscore the importance of strengthening public awareness and preventive strategies alongside biomedical screening efforts.

The Knowledge, Attitudes, and Practices (KAP) model provides a robust framework for assessing public understanding of health issues and identifying behavioral barriers to prevention and early detection. According to KAP theory, behavior change follows a sequential process: acquiring knowledge, shaping attitudes and beliefs, and ultimately adopting practices or behaviors. Notably, knowledge acquisition alone does not directly lead to behavior change; it first influences perceptions, which then drive behavioral adjustments (11). Previous research has demonstrated that increasing public knowledge about cancer significantly enhances screening participation and health-seeking behaviors (12). However, KAP studies specifically addressing NPC remain scarce, particularly in high-incidence regions such as southern China. Most existing literature focuses on clinical aspects or risk factor analysis among diagnosed patients, while community awareness, risk perception, and preventive practices among the general population remain poorly characterized. Therefore, assessing public understanding of NPC through KAP studies is essential for reducing the disease burden in high-incidence regions. This study aims to evaluate the KAP related to NPC among young adults and students in southern China, as an accessible and important subgroup for understanding health perceptions and behaviors.

## Methods

### Study design and participants

This cross-sectional study was conducted at Xiangnan University Affiliated Hospital between February 6 and March 5, 2025. Young adults and students residing in southern China were invited to participate in a questionnaire survey via an online link distributed through social media platforms (including WeChat groups and Moments) and community networks. The study received ethical approval from the Ethics Committee of Xiangnan University Affiliated Hospital (Approval No.: K2025-002-01), and informed consent was obtained from all participants. Because the survey was distributed electronically, individuals who were more familiar with online platforms may have been more likely to participate.

### Questionnaire design

The questionnaire was designed based on relevant guidelines (13). To ensure its scientific rigor and validity, a panel of 8 experts was convened to review and validate its content. Based on the panel’s feedback and recommendations, multiple revisions were made to enhance the clarity, relevance, and comprehensiveness of the questionnaire items. Following the completion of the initial draft, a small-scale pilot survey was conducted, yielding 34 valid responses. Reliability analysis demonstrated strong internal consistency, with a Cronbach’s α coefficient of 0.923, indicating high reliability.

The final questionnaire comprised 47 items across four dimensions. The demographic information section included 17 items. The knowledge dimension consisted of 12 items, encompassing true/false, single-choice, and multiple-choice questions. True/false and single-choice questions were scored as 1 point for correct answers and 0 points for incorrect or “not sure” responses. Multiple-choice questions were awarded 2 points for selecting all correct options, 1 point for partial correctness, and 0 points for incorrect answers, with a total score range of 0 to 15. The attitude dimension included 8 items, assessed using a five-point Likert scale from “Strongly agree” (5 points) to “Strongly disagree” (1 point), with a total score range of 8 to 40. The practice dimension comprised 10 items, also evaluated on a five-point Likert scale from “Very consistent” (5 points) to “Very inconsistent” (1 point), with a total score range of 10 to 50.

### Data collection and quality control

The research team trained 10 student investigators and 10 professional staff to assist in administering the standardized questionnaire. The survey platform was configured to allow only one submission per IP address to minimize duplicate entries. Additionally, respondents were required to complete all items before submission, and incomplete questionnaires could not be submitted. Data were collected electronically via the Questionarystar platform (https://www.wjx.cn). To ensure data quality, all responses were reviewed for completeness, internal logic, and consistency. Questionnaires were excluded if the completion time was less than 90 seconds, if logical inconsistencies were identified, or if responses in any KAP section were identical across all items. Because the survey link was disseminated through open online channels without a controlled sampling frame, the exact number of individuals who viewed the link could not be determined. Therefore, view rate and participation rate could not be precisely calculated.

### Sample size

The required sample size for this study was estimated using the standard formula for cross-sectional survey designs:


$$\text{n}={\left(\frac{{\text{Z}}_{1-\frac{\text{α}}{2}}}{\text{δ}}\right)}^{2}\times \text{p}\times (1-\text{p})$$

where *n* represents the required sample size, *Z_1_-α/_2_* corresponds to the critical value of the standard normal distribution for a two-tailed test, *p* denotes the anticipated prevalence of the primary outcome, and *δ* represents the allowable margin of error. Based on an expected prevalence of 50%, a 95% confidence level, and a margin of error of 5%, the calculated minimum sample size was 384 participants. To account for potential invalid or incomplete responses, an additional 20% was added to the target, resulting in a final planned sample size of approximately 480 participants to ensure the robustness and reliability of the dataset.

### Statistical analysis

Statistical analyses were conducted using R software version 4.3.2 (The R Foundation for Statistical Computing, Vienna, Austria) and Stata version 18.0 (StataCorp LLC, College Station, TX, USA). The normality of KAP score distributions was assessed using the Shapiro–Wilk or Kolmogorov–Smirnov test. Normally distributed continuous variables were reported as mean ± standard deviation (SD), with group comparisons performed using independent samples t-tests or one-way ANOVA, and Levene’s test was used to assess homogeneity of variance. Skewed continuous variables were summarized using median and interquartile range (IQR), with group comparisons conducted using the Wilcoxon–Mann–Whitney test (two groups) or Kruskal–Wallis test (three or more groups). Categorical variables were expressed as frequencies and percentages (n, %). Correlations among KAP scores were evaluated using Pearson or Spearman correlation coefficients, depending on data distribution.

Structural equation modeling (SEM) was employed to examine direct and indirect effects among knowledge, attitude, and practice dimensions, with attitude assessed as a potential mediator. The model fit was assessed using Root Mean Square Error of Approximation (RMSEA < 0.08), Standardized Root Mean Square Residual (SRMR < 0.08), Tucker-Lewis Index (TLI > 0.80), and Comparative Fit Index (CFI > 0.80). A two-sided P-value < 0.05 was considered statistically significant.

## Results

### Demographic information

Initially, a total of 766 samples were collected. The following samples were excluded: 33cases without informed consent; 16 cases with abnormal age values; 10 cases with abnormal height and weight entries; 21 cases with logically incorrect responses to quality control questions; 22 cases with a completion time of less than 90 seconds; Resulting in 664 valid responses, with an effective rate of 86.68%.

Among them, 421 (63.4%) were female, 253 (38.1%) were over 30 years old, 368 (55.4%) had a bachelor’s degree, 377 (56.8%) had a personal monthly income of less than 3,000 yuan, 315 (47.4%) had suburban residents’ medical insurance, 365 (55.0%) resided in the urban area, and 335 (50.5%) were students. The scores for knowledge, attitude, and practice were 7.99 ± 3.80 (range: 0–15), 34.45 ± 4.85 (range: 8–40), and 43.88 ± 7.30 (range: 10–50), respectively. Demographic analyses revealed significant variations in KAP scores across multiple factors. Knowledge scores differed significantly by all examined variables (medical insurance, residence, occupation, gender, age, BMI, education, marital status, smoking, and drinking; all P < 0.05). Attitude scores varied by insurance type, residence, occupation, gender, and income (P < 0.05). Practice scores showed significant associations with insurance, residence, occupation, education, income, and dietary preference (P < 0.05), while the differences related to environmental exposure were statistically marginal and small in magnitude. The most robust associations (P < 0.001) were observed between knowledge scores and insurance type, residence, age, education, and marital status (**Table 1**).

**Table 1:** Demographic characteristics and KAP scores.

N=664	N(%)	Knowledge		Attitude		Practice	
		Mean ± SD	P	Mean ± SD	P	Mean ± SD	P
Total score	664 (100.0)	7.99 ± 3.80		34.45 ± 4.85		43.88 ± 7.30	
Gender			**< 0.001**		**0.013**		0.080
Male	243 (36.6)	7.03 ± 3.93		33.72 ± 5.39		42.83 ± 8.59	
Female	421 (63.4)	8.54 ± 3.61		34.87 ± 4.46		44.49 ± 6.36	
Age			**< 0.001**		0.596		0.547
18-20	251 (37.8)	8.97 ± 3.39		34.26 ± 4.97		43.71 ± 7.81	
21-30	160 (24.1)	8.43 ± 3.41		34.32 ± 4.98		44.21 ± 7.14	
> 30	253 (38.1)	6.73 ± 4.07		34.72 ± 4.63		43.84 ± 6.88	
BMI			**0.004**		0.568		0.370
< 18.5	69 (10.4)	8.74 ± 3.60		34.75 ± 5.08		43.86 ± 7.61	
18.5-23.9	378 (56.9)	8.26 ± 3.77		34.34 ± 4.77		43.65 ± 7.01	
24.0-27.9	159 (23.9)	7.11 ± 3.84		34.60 ± 5.06		44.14 ± 8.05	
≥ 28.0	58 (8.7)	7.69 ± 3.74		34.40 ± 4.57		44.71 ± 6.63	
Education			**< 0.001**		0.071		**0.011**
Senior high school or below	112 (16.9)	4.69 ± 3.60		33.94 ± 4.35		42.64 ± 7.53	
Associate degree	184 (27.7)	8.52 ± 3.50		34.60 ± 5.51		44.26 ± 8.04	
Bachelor’s degree	368 (55.4)	8.72 ± 3.47		34.53 ± 4.63		44.07 ± 6.80	
Marital status			**< 0.001**		0.251		0.431
Single/other	415 (62.5)	8.59 ± 3.56		34.27 ± 4.98		43.86 ± 7.51	
Married	249 (37.5)	6.98 ± 3.97		34.75 ± 4.61		43.91 ± 6.94	
Personal monthly income, CNY			0.260		**0.004**		**0.001**
< 3000	377 (56.8)	8.14 ± 3.88		33.94 ± 5.18		43.33 ± 8.04	
3000-10000	247 (37.3)	7.78 ± 3.60		35.00 ± 4.24		44.20 ± 6.08	
> 10000	40 (6.0)	7.78 ± 4.22		35.77 ± 4.57		47.02 ± 6.00	
Type of medical insurance			**< 0.001**		**< 0.001**		**0.001**
Urban employees’ medical insurance	246 (37.1)	8.36 ± 3.62		35.25 ± 4.32		44.50 ± 6.30	
Suburban residents’ medical insurance	315 (47.4)	8.53 ± 3.54		34.44 ± 4.50		44.43 ± 6.72	
New rural cooperative medical insurance	79 (11.9)	5.01 ± 3.98		33.38 ± 5.68		42.00 ± 8.66	
No medical insurance	24 (3.6)	6.79 ± 3.76		29.92 ± 7.77		36.46 ± 12.78	
Place of residence			**< 0.001**		**0.020**		**0.001**
Urban	365 (55.0)	8.50 ± 3.60		34.86 ± 4.55		44.77 ± 6.72	
Suburban/rural	299 (45.0)	7.36 ± 3.95		33.95 ± 5.15		42.80 ± 7.81	
Occupation			**< 0.001**		**0.044**		**0.029**
Student	335 (50.5)	8.95 ± 3.40		34.13 ± 5.17		43.83 ± 7.80	
Company employee	58 (8.7)	7.93 ± 3.38		35.26 ± 4.11		45.72 ± 5.75	
Freelance	74 (11.1)	6.45 ± 3.82		34.51 ± 5.05		43.91 ± 6.91	
Worker	19 (2.9)	5.58 ± 3.91		34.37 ± 4.10		41.42 ± 7.25	
Teacher	105 (15.8)	7.61 ± 3.59		34.71 ± 4.38		43.46 ± 6.33	
Civil servant	5 (0.8)	7.20 ± 3.90		35.60 ± 3.58		47.60 ± 4.34	
Retired or unemployed	28 (4.2)	3.21 ± 3.36		34.29 ± 4.41		42.54 ± 8.34	
Doctor	10 (1.5)	10.10 ± 3.63		36.80 ± 3.61		47.20 ± 2.97	
Nurse	11(1.7)	8.73 ± 4.03		35.18 ± 5.29		44.27 ± 9.34	
Medical technician	7(1.1)	12.43 ± 1.51		38.29 ± 1.80		47.14 ± 5.24	
Other	12(1.8)	4.42 ± 3.55		31.83 ± 4.11		40.25 ± 7.66	
Smoking			**0.003**		0.482		0.087
No	576(86.7)	8.16 ± 3.75		34.51 ± 4.80		44.12 ± 7.10	
Yes	88(13.3)	6.85 ± 3.93		34.03 ± 5.13		42.32 ± 8.32	
Drinking			**0.013**		0.446		0.091
No	543(81.8)	8.16 ± 3.77		34.51 ± 4.83		44.12 ± 7.18	
Yes	121(18.2)	7.22 ± 3.87		34.17 ± 4.93		42.80 ± 7.75	
History of nasopharyngeal carcinoma			0.067		0.063		0.273
No	659(99.2)	8.00 ± 3.81		34.49 ± 4.81		43.93 ± 7.23	
Yes	5(0.8)	5.60 ± 0.89		29.00 ± 7.28		37.40 ± 12.99	
Consume pickled food			0.827		0.089		**0.046**
No	521(78.5)	8.01 ± 3.80		34.57 ± 4.87		44.13 ± 7.25	
Yes	143(21.5)	7.91 ± 3.83		34.00 ± 4.75		42.97 ± 7.41	
Environmental pollution within 500m of your residence			0.248		0.287		**0.046**
No	633(95.3)	8.02 ± 3.80		34.49 ± 4.84		43.99 ± 7.31	
Yes	31(4.7)	7.39 ± 3.84		33.58 ± 4.94		41.68 ± 6.84	
Environmental pollution within 500m of your workplace			0.281		0.519		0.250
No	633(95.3)	8.01 ± 3.81		34.48 ± 4.83		43.98 ± 7.18	
Yes	31(4.7)	7.52 ± 3.51		33.90 ± 5.28		41.77 ± 9.34	

Bold values indicate statistically significant differences (P < 0.05).

### Distribution of responses to knowledge, attitude, and practice

The distribution of knowledge dimension revealed significant gaps in participants’ knowledge regarding NPC. The item with the highest correct response rate was “smoking is not related to NPC” (65.96% correctly disagreed), while the lowest was “pickled foods are unrelated to NPC,” with only 16.42% correctly identifying the dietary risk. Although over half of participants were aware that NPC is a common head and neck malignancy, substantial misconceptions remained regarding its etiology, particularly concerning EBV infection (only 43.83% correct) and dietary risks such as pickled foods (16.42% correct). Confusion about clinical presentations was also evident, with 37.95% incorrectly selecting abdominal pain as an early symptom and 31.93% attributing NPC to unrelated factors such as climate change. In terms of prevention and management, less than half of the participants correctly identified EBV avoidance and family nbsp; history as key factors, and awareness of standard treatments—such as radiotherapy, chemotherapy, and targeted therapy—was similarly limited. These findings underscore substantial gaps in understanding NPC etiology, prevention, and management, particularly concerning EBV, dietary risks, and modern treatment modalities (**Supplementary Table 1**).

Responses to the attitude dimension showed that 19.58% strongly agreed and 11.3% agreed that nasopharyngeal carcinoma is rare and therefore does not require much attention (A7). Meanwhile, only 48.19% strongly agreed that nasopharyngeal carcinoma is related to EB virus infection (A4) (**Supplementary Table 2**).

Responses to the practice dimension showed that only 48.64% strongly agreed that they would undergo regular health check-ups to detect nasopharyngeal carcinoma early (P1) and only 49.1% strongly agreed that they would pay attention to reducing the intake of pickled foods in their daily diet (P2) (**Supplementary Table 3**).

### Correlations analysis

Further correlation analysis revealed positive correlations between knowledge scores and attitude scores (r = 0.353, P < 0.001), as well as between knowledge scores and practice scores (r = 0.247, P < 0.001). Additionally, attitude scores were positively correlated with practice scores (r = 0.679, P < 0.001) (**Table 2**).

**Table 2:** Correlation analysis.

Spearman	Knowledge	Attitude	Practice
Knowledge	1.000		
Attitude	0.353 (P<0.001)	1.000	
Practice	0.247 (P<0.001)	0.679 (P<0.001)	1.000

### SEM analysis

**Figure 1:**
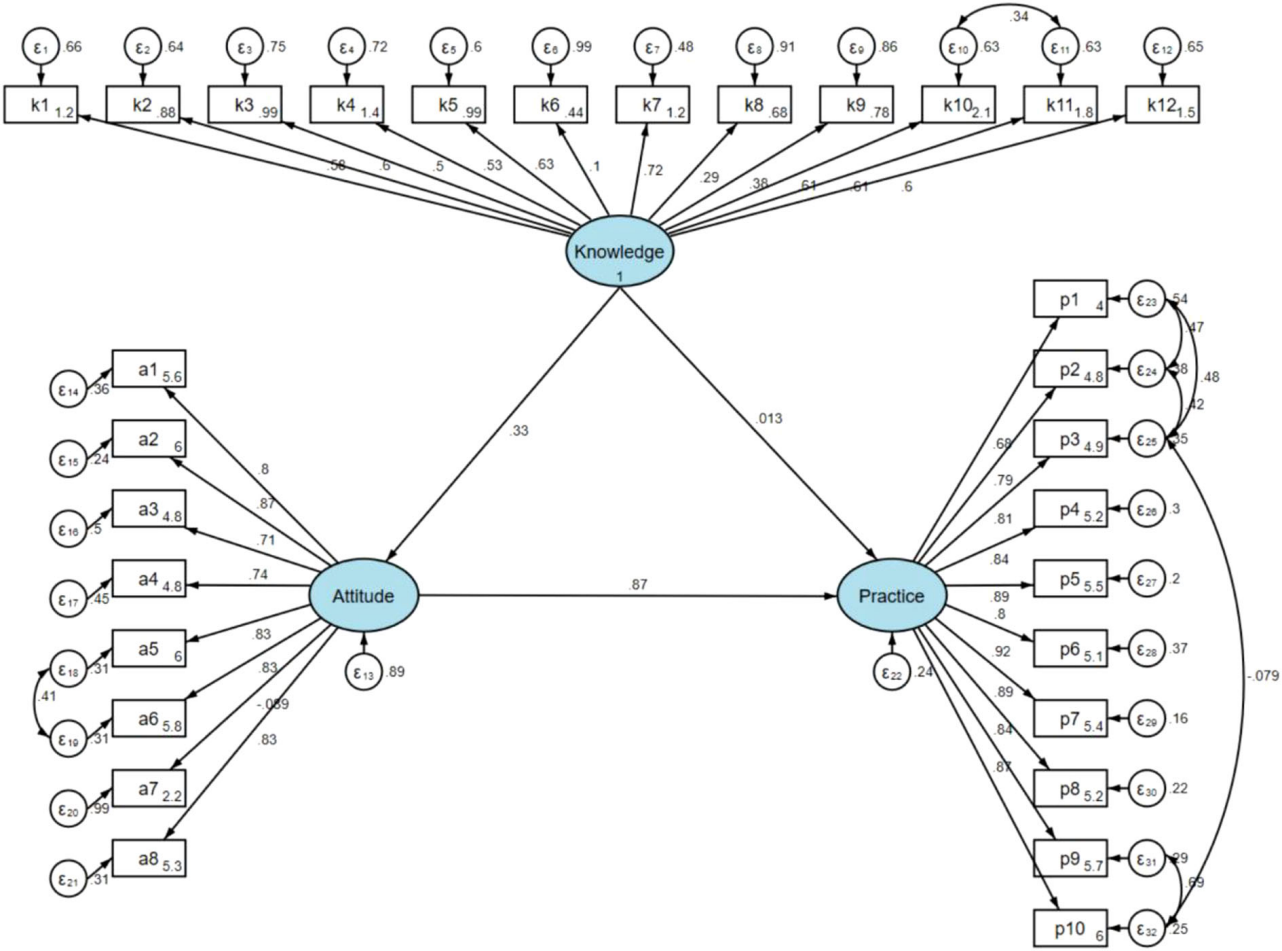
SEM model.

The fit of the SEM model yielded good indices demonstrating good model fit (RMSEA value: 0.059, SRMR value: 0.047, TLI value: 0.927, and CFI value: 0.933) (**Supplementary Table 4**). The results of direct and indirect effects showed that the direct effect of knowledge on attitude (β = 0.329, P < 0.001), as well as of attitude on practice (β = 0.866, P < 0.001). It is worth noting that while knowledge does not directly affect practice, it has an indirect effect on practice through attitude (β = 0.285, P < 0.001) (**Table 3** and **Figure 1**).

**Table 3:** SEM analysis.

Model paths	Total effects		Direct effect		Indirect effect	
	β (95%CI)	P	β (95%CI)	P	β (95%CI)	P
Attitude						
Knowledge	0.329 (0.250, 0.407)	< 0.001	0.329 (0.250, 0.407)	< 0.001		
Practice						
Knowledge	0.297 (0.219, 0.375)	< 0.001	0.013 (-0.040, 0.065)	0.641	0.285 (0.215, 0.354)	< 0.001
Attitude	0.866 (0.836, 0.0.897)	< 0.001	0.866 (0.836, 0.0.897)	< 0.001		

## Discussion

Young adults and students in southern China demonstrated insufficient knowledge yet reported relatively positive attitudes and proactive practices related to NPC. This apparent discrepancy warrants careful interpretation. The practice dimension primarily reflects self-reported behavioral tendencies and general health-related actions rather than objectively verified NPC-specific preventive behaviors. Therefore, reported “good practice” may partly represent general health awareness or socially desirable responses rather than behavior grounded in accurate disease-specific knowledge. These findings highlight the need for targeted health education initiatives aimed at enhancing public knowledge, which may further strengthen evidence-based preventive behaviors through the reinforcement of positive attitudes.

The pattern of insufficient knowledge despite favorable attitudes and behaviors echoes trends reported in cancer-related KAP and awareness studies, where positive health behaviors are often driven by generalized beliefs in health maintenance rather than specific disease awareness (14–16). However, consistent with prior NPC-focused literature, basic epidemiological knowledge—such as the role of Epstein–Barr virus infection, dietary risk factors, and early symptom recognition—remains inadequately disseminated (6, 10).

The relational patterns uncovered through correlation analysis and SEM further substantiate these concerns. While knowledge was moderately associated with both attitudes and practices, a much stronger link was observed between attitudes and practices. SEM results reinforced that knowledge influenced practices only indirectly through its effect on attitudes. This finding is consistent with models of health behavior that emphasize the role of attitudinal mediation in translating cognitive awareness into actionable behavior (15, 16). The relatively high standardized coefficient observed between attitude and practice may be attributable to the conceptual proximity of these two constructs, as both were measured using self-reported Likert-scale items within the same survey context. Such measurement characteristics may increase the strength of observed associations due to shared method variance. Although the SEM fit indices indicated acceptable model fit, the magnitude of this path should be interpreted with consideration of potential construct overlap and common method effects. However, it is also possible that the practice items captured behavioral intention or self-perceived compliance rather than objectively measured preventive actions, which may partially explain the relatively high practice scores despite limited knowledge. Studies in similar populations have similarly suggested that without fostering the emotional and motivational components reflected in attitudes, the effect of factual knowledge on behavior change remains limited (17).

Gender differences, with women exhibiting comparatively higher knowledge and attitude scores, are consistent with evidence indicating that women tend to be more engaged with online health information seeking and preventive healthcare use (18, 19). Age-related patterns showed that younger individuals possessed better knowledge but not necessarily more proactive practices, which mirrors findings from studies emphasizing that youth are often better informed due to educational exposure yet may lack sustained preventive behavior (20). Differences based on educational attainment were pronounced, affirming the broader literature that associates higher levels of formal education with greater health literacy and cancer awareness (21). Furthermore, the urban–rural divide in knowledge and practice reflects persistent structural inequalities in healthcare access and educational resources, patterns that have been extensively documented in health disparities research within China and other low- and middle-income countries (22).

Distributional patterns across knowledge items also provide important insights. While general awareness about the importance of early detection was relatively widespread, detailed knowledge regarding risk factors such as EBV infection and dietary influences was much less consistent. Similar gaps have been identified in previous NPC-related surveys, suggesting that public health campaigns may have been more successful in promoting general cancer vigilance than in imparting specific, actionable knowledge (23). The lower levels of recognition for early symptoms such as blood-stained nasal discharge or unexplained tinnitus are particularly concerning given that delayed symptom recognition is a major contributor to late-stage NPC diagnoses in high-burden regions.

Attitudinal distributions presented a relatively optimistic picture, with the majority of respondents expressing strong beliefs in the importance of early detection and healthy lifestyles. However, a substantial proportion also viewed NPC as a rare condition, implying a misperception of personal risk. Studies in this field have suggested that the perceived rarity of certain cancers leads to lower prioritization of screening and preventive actions, even in high-incidence regions (24, 25). This misalignment between objective risk and subjective perception has been documented in other high-burden diseases, where familiarity with the disease at the community level does not always correlate with accurate personal risk appraisal (26).

In terms of practice patterns, while many participants reported engaging in preventive behaviors, practices directly related to specific modifiable NPC risk factors—such as reducing intake of pickled foods—were less consistently grounded in accurate knowledge. Notably, although only a minority correctly identified pickled foods as a risk factor in the knowledge dimension, a larger proportion reported attempting to reduce such intake. This suggests that reported practices may reflect general health consciousness rather than explicit understanding of NPC-related risks. Similar patterns have been observed in broader public health research, where individuals more readily endorse generalized health behaviors than behaviors directly informed by disease-specific knowledge (27). The relative difficulty in modifying dietary and smoking behaviors points to a broader challenge in translating awareness and positive attitudes into substantive lifestyle changes, an issue frequently noted in chronic disease prevention efforts (28).

Socio-cultural factors, healthcare system characteristics, and resource distribution disparities appear to substantially shape these patterns. Studies in comparable settings have shown that urban residents generally benefit from more consistent health promotion efforts and greater healthcare service access, whereas rural and suburban populations often experience fragmented service delivery and lower health literacy exposure (29). Furthermore, the structure of primary healthcare services and the extent of their integration with public health initiatives significantly influence whether knowledge translates into preventive behavior (30). In regions where preventive services are underfunded or poorly coordinated, even populations with relatively high health awareness may exhibit gaps in practice adoption (31).

Given these findings, several recommendations emerge. Fundamentally, healthcare systems must move toward integrating NPC-specific health education into routine community health services, particularly focusing on rural and suburban areas where gaps are more pronounced. Rather than solely disseminating information, initiatives should focus on building risk awareness and correcting misconceptions about disease rarity. Interventions such as community-based participatory education programs, which have shown success in other settings by leveraging local networks and culturally adapted messaging, could prove effective (32).

Educational interventions should also move beyond traditional mass media approaches. Collaborations between healthcare providers, educational institutions, and community organizations could facilitate tailored workshops and campaigns that emphasize early symptom recognition, risk factor modification, and the importance of screening in high-incidence areas. Drawing on evidence from similar regional interventions, training programs targeting healthcare professionals in primary care settings to deliver brief, opportunistic cancer prevention counseling during routine visits could be highly impactful (33).

However, several limitations should be acknowledged. First, as a cross-sectional study, causal relationships among knowledge, attitudes, and practices could not be definitively established. Second, the use of self-reported questionnaires may have introduced response bias, potentially overestimating positive attitudes and practices. Third, although participants were recruited from the general population through online dissemination, the use of an electronic questionnaire may have led to a higher participation rate among younger and more educated individuals who are more familiar with digital platforms. Fourth, and most critically, our sample was predominantly composed of young individuals (61.9% under 30 years old) and students (50.5%), which limits the generalizability of our findings to the broader adult population in Southern China, particularly older adults and individuals in non-student occupations. The findings should therefore be interpreted as primarily reflecting the KAP status of younger and student subgroups. Therefore, the demographic distribution of the sample may not fully represent the entire population of Southern China, which may limit the generalizability of the findings.

## Conclusion

In conclusion, young adults and students in Southern China exhibit low knowledge of NPC but report positive attitudes and practices. Future targeted health education interventions for this demographic could enhance their knowledge, thereby further strengthening positive attitudes and promoting more effective preventive behaviors in clinical and community settings.

## Data Availability

The original contributions presented in the study are included in the article/**Supplementary Material**. Further inquiries can be directed to the corresponding author/s.
