# Supplementary material for: Knowledge, attitudes, and practices regarding nasopharyngeal carcinoma among young adults and students in southern China: a cross-sectional study
Source: Front Oncol. 2026 Mar 19;16:1776276. doi: 10.3389/fonc.2026.1776276 (PMC13043359; doi:10.3389/fonc.2026.1776276)
Supplement: Supplementary file 1 [file DataSheet1.docx]

| **Survey on the Knowledge, Attitudes, and Practices Regarding Nasopharyngeal Carcinoma Among the General Population**  Questionnaire ID: | | | |
| --- | --- | --- | --- |
| Dear Participant,  Greetings! We are a research team conducting a study on the knowledge, attitudes, and practices related to nasopharyngeal carcinoma (NPC). We sincerely invite you to participate in this study. The purpose of this research is to understand your awareness, perceptions, and related behaviors concerning NPC in your daily life. The findings will help us develop evidence-based intervention strategies that may benefit more people and improve public health outcomes. Your participation is completely voluntary. This study has been reviewed and approved by the Ethics Committee. If you agree to participate, please refer to the following instructions:  1. Please complete the questionnaire. There are no right or wrong answers; simply answer based on your actual situation. If you encounter any issues during the process, feel free to contact us. After completion, please submit the questionnaire promptly.  2. This is a simple survey and will not cause any harm to your physical or mental health. However, it does involve some private information, such as your gender and age. We will keep all information strictly confidential and ensure your privacy.  3. As a participant, you are entitled to request information about the study and its progress at any time. If you decide to withdraw from the study, please inform us. Your data will not be included in the analysis.  Thank you very much for taking the time out of your busy schedule to support our scientific research!  □I have been informed and agree that the collected data will be used for scientific research.  Informed Consent Signature:  Date of Participation: _____ / _____ / _____ (YYYY/MM/DD) | | | |
| **Part 1 Basic Information** | | |  |
| 1. **Your gender:** | A. Male | B. Female |  |
| 1. **Your age: _____ years old** | | |  |
| 1. **Your weight: _____ kg** | | |  |
| 1. **Your height: _____ cm** | | |  |
| 1. **Your education level:** | A. Primary school or below B. Junior high school C. Senior high school / Technical secondary school D. Associate/ Bachelor's degree or above | |  |
| 1. **Your marital status:** | A. Single B. Married C. Divorced D. Widowed E. Separated F. Other, please specify: __________ | |  |
| 1. **Your monthly personal income，CNY:** | A. Below 3,000  B. 3,000–4,999  C. 5,000–6,999  D. 7,000–10,000  E. Above 10,000 | |  |
| 1. **Your medical insurance type:** | A. Urban Employee Medical Insurance B. Urban and Rural Resident Medical Insurance C. New Rural Cooperative Medical Insurance D. Commercial Medical Insurance E. No Medical Insurance | |  |
| 1. **Your place of residence:** | A. Urban B. Suburban C. Rural | |  |
| 1. **Your province of residence:** | Province | |  |
| 1. **Your current occupation:** | A. Student B. Company employee C. Freelancer D. Worker E. Teacher F. Civil servant G. Retired / Unemployed H. Doctor I. Nurse J. Medical technician K. Other, please specify: __________ | |  |
| 1. **Do you smoke?** | 1. Yes 2. No | |  |
| 1. **Do you consume alcohol?** | 1. Yes 2. No | |  |
| 1. **Do you or your immediate family members have a history of nasopharyngeal carcinoma?** | 1. Yes 2. No | |  |
| 1. **Do you frequently consume pickled foods?** | 1. Yes 2. No | |  |
| 1. **Is there any environmental pollution (e.g., chemical plants, coal mines, stone processing sites) within 500 meters of your residence?** | 1. Yes 2. No | |  |
| 1. **Is there any environmental pollution (e.g., chemical plants, coal mines, stone processing sites) within 500 meters of your workplace?** | 1. Yes 2. No | |  |

**Part 2 Knowledge Dimension on Nasopharyngeal Carcinoma**

Please select the option that best reflects your understanding of each statement. If you are unsure about an answer, please choose "Not sure."

| **True or False Questions** | |
| --- | --- |
| 1. Nasopharyngeal carcinoma is a common malignant tumor of the head and neck. | A. True  B. False  C. Not sure |
| 1. Nasopharyngeal carcinoma is associated with Epstein-Barr virus (EBV) infection. | A. True  B. False  C. Not sure |
| 1. Males are more likely than females to develop nasopharyngeal carcinoma. | A. True  B. False  C. Not sure |
| 1. Smoking is not related to nasopharyngeal carcinoma. | A. True  B. False  C. Not sure |
| 1. Nasopharyngeal carcinoma has a high incidence in southern and southeastern coastal regions of China. | A. True  B. False  C. Not sure |
| 1. Pickled foods in the diet are not related to the development of nasopharyngeal carcinoma. | A. True  B. False  C. Not sure |
| 1. Patients with nasopharyngeal carcinoma often present with symptoms of enlarged cervical lymph nodes. | A. True  B. False  C. Not sure |
| 1. Shanghai is the capital of China. | A. True  B. False  C. Not sure |
| **Single Choice Questions** | |
| 1. Which of the following is NOT associated with the development of nasopharyngeal carcinoma? | A. EBV infection B. High-salt diet C. Climate change D. Family history E. Not sure |
| 1. Which of the following is NOT a common early symptom of nasopharyngeal carcinoma? | A. Nasal congestion B. Nosebleeds C. Blurred vision D. Abdominal pain E. Tinnitus or hearing loss F. Not sure |
| **Multiple Choice Questions** | |
| 1. Which of the following factors may help prevent nasopharyngeal carcinoma? (Select all that apply) | A. Improving dietary habits B. Quitting smoking C. Actively avoiding or staying away from secondhand smoke D. Avoiding EBV infection E. Increasing physical activity F. Not sure |
| 1. Under which of the following circumstances should screening for nasopharyngeal carcinoma be considered? (Select all that apply) | A. Family history of the disease B. Tinnitus or hearing loss C. Blood-stained nasal discharge D. Long-term nasal congestion with no other identifiable cause E. Numbness or headache without obvious cause F. Enlarged cervical lymph nodes G. Not sure |
| 1. What are the treatment methods for nasopharyngeal carcinoma? (Select all that apply) | A. Surgery B. Radiotherapy C. Chemotherapy D. Combined radiotherapy and chemotherapy E. Targeted therapy F. Immunotherapy G. Not sure |

| **Part 3 Attitude Dimension on Nasopharyngeal Carcinoma**  Please select one option from “Strongly agree” to “Strongly disagree” based on whether you agree with the following statements. | | | | | |
| --- | --- | --- | --- | --- | --- |
| 1. I think it is very important to understand the early symptoms of nasopharyngeal carcinoma. | a. strongly agree | b. agree | c. neutral | d. disagree | e. strongly disagree |
| 1. I believe nasopharyngeal carcinoma can be prevented through a healthy lifestyle. | a. strongly agree | b. agree | c. neutral | d. disagree | e. strongly disagree |
| 1. I think nasopharyngeal carcinoma is related to air pollution. | a. strongly agree | b. agree | c. neutral | d. disagree | e. strongly disagree |
| 1. I think nasopharyngeal carcinoma is related to EB virus infection. | a. strongly agree | b. agree | c. neutral | d. disagree | e. strongly disagree |
| 1. I believe that early detection of nasopharyngeal carcinoma contributes to better treatment outcomes and prognosis. | a. strongly agree | b. agree | c. neutral | d. disagree | e. strongly disagree |
| 1. If I have a family history of the disease, I will pay more attention to the prevention of nasopharyngeal carcinoma. | a. strongly agree | b. agree | c. neutral | d. disagree | e. strongly disagree |
| 1. I think nasopharyngeal carcinoma is rare and therefore does not require much attention. | a. strongly agree | b. agree | c. neutral | d. disagree | e. strongly disagree |
| 1. Participating in health check-ups related to nasopharyngeal carcinoma is important to me. | a. strongly agree | b. agree | c. neutral | d. disagree | e. strongly disagree |
| **Part 4 Behavioral Practices on Nasopharyngeal Carcinoma**  Please select one option from “Strongly agree” to “Strongly disagree” based on whether the following statements describe your behavior. | | | | | |
| 1. I undergo regular health check-ups to detect nasopharyngeal carcinoma early. | a. strongly agree | b. agree | c. neutral | d. disagree | e. strongly disagree |
| 1. I pay attention to reducing the intake of pickled foods in my daily diet. | a. strongly agree | b. agree | c. neutral | d. disagree | e. strongly disagree |
| 1. I advise my family or friends to undergo regular health check-ups to rule out nasopharyngeal carcinoma and other diseases. | a. strongly agree | b. agree | c. neutral | d. disagree | e. strongly disagree |
| 1. If family members or friends have symptoms such as blood-streaked nasal discharge, tinnitus, or nasal congestion, I will recommend that they seek medical attention promptly. | a. strongly agree | b. agree | c. neutral | d. disagree | e. strongly disagree |
| 1. I try to avoid smoking to reduce the risk of nasopharyngeal carcinoma. | a. strongly agree | b. agree | c. neutral | d. disagree | e. strongly disagree |
| 1. I actively avoid inhaling secondhand smoke to reduce the risk of nasopharyngeal carcinoma. | a. strongly agree | b. agree | c. neutral | d. disagree | e. strongly disagree |
| 1. I pay attention to air quality in daily life and take protective measures to reduce the risk of nasopharyngeal carcinoma. | a. strongly agree | b. agree | c. neutral | d. disagree | e. strongly disagree |
| 1. I encourage my family and friends to pay attention to the prevention of nasopharyngeal carcinoma. | a. strongly agree | b. agree | c. neutral | d. disagree | e. strongly disagree |
| 1. If I were diagnosed with nasopharyngeal carcinoma, I would actively cooperate with doctors for treatment. | a. strongly agree | b. agree | c. neutral | d. disagree | e. strongly disagree |
| 1. If a friend or family member were diagnosed with nasopharyngeal carcinoma, I would encourage them to actively cooperate with doctors for treatment. | a. strongly agree | b. agree | c. neutral | d. disagree | e. strongly disagree |
