# Supplementary material for: Knowledge, attitudes, and practices regarding nasopharyngeal carcinoma among young adults and students in southern China: a cross-sectional study
Source: Front Oncol. 2026 Mar 19;16:1776276. doi: 10.3389/fonc.2026.1776276 (PMC13043359; doi:10.3389/fonc.2026.1776276)
Supplement: Supplementary file 2 [file Table1.docx]

**Supplementary table 1. Distribution of knowledge dimension responses**

| Knowledge | Option a | Option b | Option c | Option d | Option e | Option f | Average score |
| --- | --- | --- | --- | --- | --- | --- | --- |
|  | True | False | Not sure |  |  |  |  |
| 1. Nasopharyngeal carcinoma is a common malignant tumor of the head and neck. | 396 (59.64%) | 65 (9.79%) | 203 (30.57%) |  |  |  | 0.60 (0.49) |
| 2. Nasopharyngeal carcinoma is associated with Epstein-Barr virus (EBV) infection. | 291 (43.83%) | 43 (6.48%) | 330 (49.7%) |  |  |  | 0.44 (0.50) |
| 3. Males are more likely than females to develop nasopharyngeal carcinoma. | 327 (49.25%) | 47 (7.08%) | 290 (43.67%) |  |  |  | 0.49 (0.50) |
| 4. Smoking is not related to nasopharyngeal carcinoma. | 85 (12.8%) | 438 (65.96%) | 141 (21.23%) |  |  |  | 0.66 (0.47) |
| 5. Nasopharyngeal carcinoma has a high incidence in southern and southeastern coastal regions of China. | 327 (49.25%) | 53 (7.98%) | 284 (42.77%) |  |  |  | 0.49 (0.50) |
| 6. Pickled foods in the diet are not related to the development of nasopharyngeal carcinoma. | 109 (16.42%) | 387 (58.28%) | 168 (25.3%) |  |  |  | 0.16 (0.37) |
| 7. Patients with nasopharyngeal carcinoma often present with symptoms of enlarged cervical lymph nodes. | 403 (60.69%) | 33 (4.97%) | 228 (34.34%) |  |  |  | 0.61 (0.49) |
|  | EBV infection | High-salt diet | Climate change | Family history | Not sure |  |  |
| 9. Which of the following is NOT associated with the development of nasopharyngeal carcinoma? | 59 (8.89%) | 62 (9.34%) | 212 (31.93%) | 65 (9.79%) | 266 (40.06%) |  | 0.32 (0.47) |
|  | A. Nasal congestion | B. Nosebleeds | C. Blurred vision | D. Abdominal pain | E. Tinnitus or hearing loss | F. Not sure |  |
| 10. Which of the following is NOT a common early symptom of nasopharyngeal carcinoma? | 47 (7.08%) | 30 (4.52%) | 71 (10.69%) | 252 (37.95%) | 26 (3.92%) | 238 (35.84%) | 0.38 (0.49) |
|  | Select this option | Not select this option |  |  |  |  |  |
| 11. Which of the following factors may help prevent nasopharyngeal carcinoma? (Select all that apply)   1. Improving dietary habits | 546 (82.23%) | 118 (17.77%) |  |  |  |  | 1.47 (0.71) |
| B. Quitting smoking | 530 (79.82%) | 134 (20.18%) |  |  |  |  |  |
| C. Actively avoiding or staying away from secondhand smoke | 531 (79.97%) | 133 (20.03%) |  |  |  |  |  |
| D. Avoiding EBV infection | 477 (71.84%) | 187 (28.16%) |  |  |  |  |  |
| E. Increasing physical activity | 472 (71.08%) | 192 (28.92%) |  |  |  |  |  |
| F. Not sure | 83 (12.5%) | 581 (87.5%) |  |  |  |  |  |
| 12. Which of the following circumstances should screening for nasopharyngeal carcinoma be considered? (Select all that apply)  A. Family history of the disease | 467 (70.33%) | 197 (29.67%) |  |  |  |  | 1.25 (0.70) |
| B. Tinnitus or hearing loss | 400 (60.24%) | 264 (39.76%) |  |  |  |  |  |
| C. Blood-stained nasal discharge | 493 (74.25%) | 171 (25.75%) |  |  |  |  |  |
| D. Long-term nasal congestion with no other identifiable cause | 445 (67.02%) | 219 (32.98%) |  |  |  |  |  |
| E. Numbness or headache without obvious cause | 393 (59.19%) | 271 (40.81%) |  |  |  |  |  |
| F. Enlarged cervical lymph nodes | 423 (63.7%) | 241 (36.3%) |  |  |  |  |  |
| G. Not sure | 102 (15.36%) | 562 (84.64%) |  |  |  |  |  |
| 13. What are the treatment methods for nasopharyngeal carcinoma? (Select all that apply)  A. Surgery | 490 (73.8%) | 174 (26.2%) |  |  |  |  | 1.12 (0.75) |
| B. Radiotherapy | 377 (56.78%) | 287 (43.22%) |  |  |  |  |  |
| C. Chemotherapy | 389 (58.58%) | 275 (41.42%) |  |  |  |  |  |
| D. Combined radiotherapy and chemotherapy | 400 (60.24%) | 264 (39.76%) |  |  |  |  |  |
| E. Targeted therapy | 342 (51.51%) | 322 (48.49%) |  |  |  |  |  |
| F. Immunotherapy | 293 (44.13%) | 371 (55.87%) |  |  |  |  |  |
| G. Not sure | 154 (23.19%) | 510 (76.81%) |  |  |  |  |  |

**Supplementary table 2. Distribution of attitude dimension responses**

| **Attitude** | Strongly agree | Agree | Neutral | Disagree | Strongly disagree |
| --- | --- | --- | --- | --- | --- |
| 1. I think it is very important to understand the early symptoms of nasopharyngeal carcinoma. | 440 (66.27%) | 162 (24.4%) | 41 (6.17%) | 10 (1.51%) | 11 (1.66%) |
| 2. I believe nasopharyngeal carcinoma can be prevented through a healthy lifestyle. | 398 (59.94%) | 213 (32.08%) | 40 (6.02%) | 5 (0.75%) | 8 (1.2%) |
| 3. I think nasopharyngeal carcinoma is related to air pollution. | 344 (51.81%) | 226 (34.04%) | 65 (9.79%) | 13 (1.96%) | 16 (2.41%) |
| 4. I think nasopharyngeal carcinoma is related to EB virus infection. | 320 (48.19%) | 220 (33.13%) | 101 (15.21%) | 14 (2.11%) | 9 (1.36%) |
| 5. I believe that early detection of nasopharyngeal carcinoma contributes to better treatment outcomes and prognosis. | 424 (63.86%) | 195 (29.37%) | 28 (4.22%) | 7 (1.05%) | 10 (1.51%) |
| 6. If I have a family history of the disease, I will pay more attention to the prevention of nasopharyngeal carcinoma. | 416 (62.65%) | 194 (29.22%) | 34 (5.12%) | 12 (1.81%) | 8 (1.2%) |
| 7. I think nasopharyngeal carcinoma is rare and therefore does not require much attention. | 130 (19.58%) | 75 (11.3%) | 62 (9.34%) | 158 (23.8%) | 239 (35.99%) |
| 8. Participating in health check-ups related to nasopharyngeal carcinoma is important to me. | 377 (56.78%) | 200 (30.12%) | 67 (10.09%) | 14 (2.11%) | 6 (0.9%) |

**Supplementary table 3. Distribution of practice dimension responses**

| **Practice** | Strongly agree | Agree | Neutral | Disagree | Strongly disagree |
| --- | --- | --- | --- | --- | --- |
| 1. I undergo regular health check-ups to detect nasopharyngeal carcinoma early. | 323 (48.64%) | 181 (27.26%) | 102 (15.36%) | 43 (6.48%) | 15 (2.26%) |
| 2. I pay attention to reducing the intake of pickled foods in my daily diet. | 326 (49.1%) | 225 (33.89%) | 84 (12.65%) | 20 (3.01%) | 9 (1.36%) |
| 3. I advise my family or friends to undergo regular health check-ups to rule out nasopharyngeal carcinoma and other diseases. | 352 (53.01%) | 195 (29.37%) | 92 (13.86%) | 17 (2.56%) | 8 (1.2%) |
| 4. If family members or friends have symptoms such as blood-streaked nasal discharge, tinnitus, or nasal congestion, I will recommend that they seek medical attention promptly. | 363 (54.67%) | 212 (31.93%) | 66 (9.94%) | 15 (2.26%) | 8 (1.2%) |
| 5. I try to avoid smoking to reduce the risk of nasopharyngeal carcinoma. | 419 (63.1%) | 176 (26.51%) | 48 (7.23%) | 13 (1.96%) | 8 (1.2%) |
| 6. I actively avoid inhaling secondhand smoke to reduce the risk of nasopharyngeal carcinoma. | 408 (61.45%) | 183 (27.56%) | 47 (7.08%) | 11 (1.66%) | 15 (2.26%) |
| 7. I pay attention to air quality in daily life and take protective measures to reduce the risk of nasopharyngeal carcinoma. | 395 (59.49%) | 188 (28.31%) | 62 (9.34%) | 12 (1.81%) | 7 (1.05%) |
| 8. I encourage my family and friends to pay attention to the prevention of nasopharyngeal carcinoma. | 383 (57.68%) | 197 (29.67%) | 62 (9.34%) | 12 (1.81%) | 10 (1.51%) |
| 9. If I were diagnosed with nasopharyngeal carcinoma, I would actively cooperate with doctors for treatment. | 424 (63.86%) | 187 (28.16%) | 33 (4.97%) | 10 (1.51%) | 10 (1.51%) |
| 10. If a friend or family member were diagnosed with nasopharyngeal carcinoma, I would encourage them to actively cooperate with doctors for treatment. | 429 (64.61%) | 184 (27.71%) | 36 (5.42%) | 7 (1.05%) | 8 (1.2%) |

**Supplementary table 4. SEM fit indicators**

| **Indicators** | **Reference** | **Results** |
| --- | --- | --- |
| RMSEA | <0.080 | 0.059 |
| SRMR | <0.080 | 0.047 |
| TLI | >0.800 | 0.927 |
| CFI | >0.800 | 0.933 |
